# Calcium-activated chloride channel regulator 1 (CLCA1): More than a regulator of chloride transport and mucus production^☆^

**DOI:** 10.1016/j.waojou.2019.100077

**Published:** 2019-11-29

**Authors:** Cong-Lin Liu, Guo-Ping Shi

**Affiliations:** a Department of Cardiology, The First Affiliated Hospital of Zhengzhou University, Zhengzhou, 450000, China; b Department of Medicine, Brigham and Women's Hospital and Harvard Medical School, Boston, MA, 02115, USA

**Keywords:** CLCA1, Mucin, Innate immunity, Respiratory diseases, Gastrointestinal disease, Cancer, CLCA1, calcium-activated chloride channel regulator 1, Gob-5, goblet cell protein-5, VWA, von Willebrand factor type A, CaCCs, calcium-activated chloride channels, KCNMB1, potassium calcium-activated channel subfamily M regulatory beta subunit 1, TMEM16A, transmembrane protein 16A, IAD, inflammatory airway diseases, DOG1, discovered on gastrointestinal stromal tumours-1, COPD, chronic obstructive pulmonary disease, TNF-α, tumor necrosis factor-α, LFC, log2 fold change, HDMA, house dust mite allergen, BALF, bronchoalveolar lavage fluid, CeO_2_NPs, cerium dioxide nanoparticles, OVA, ovalbumin, MUC5AC, mucin 5AC, WT, wild-type, CF, cystic fibrosis, CFTR, cystic fibrosis transmembrane conductance regulator, cAMP, cyclic adenosine monophosphate, CXCL-1, C-X-C motif chemokine ligand 1, Bpifa1, bactericidal/permeability-increasing protein (BPI) fold-containing family A member 1, STAT6, signal transducer and activator of transcription 6, SPDEF, sterile alpha motif [SAM] domain-containing prostate-derived Ets transcription factor, EGFR, epidermal growth factor receptor, NFA, niflumic acid, EMT, epithelial-mesenchymal transition, β_4_BMs, β_4_-binding motifs, FAK, focal adhesion kinase, ERK, extracellular signal-regulated kinase, DSS, dextran sodium sulfate, rIFABP, rat intestinal fatty acid binding protein promoter, EpOCs, epithelial organoid cultures, AMCase, acidic mammalian chitinase, LFA-1, lymphocyte function-associated antigen 1.

## Abstract

CLCA1 is a member of the CLCA (calcium-activated chloride channel regulator) family and plays an essential role in goblet cell mucus production from the respiratory tract epithelium. CLCA1 also regulates Ca^2+^-dependent Cl^−^ transport that involves the channel protein transmembrane protein 16A (TMEM16A) and its accessary molecules. CLCA1 modulates epithelial cell chloride current and participates in the pathogenesis of mucus hypersecretory-associated respiratory and gastrointestinal diseases, including asthma, chronic obstructive pulmonary disease, cystic fibrosis, pneumonia, colon colitis, cystic fibrosis intestinal mucous disease, ulcerative colitis, and gastrointestinal parasitic infection. Most studies have been focused on the expression regulation of CLCA1 in human specimens. Limited studies used the CLCA1-deficient mice and CLCA1 blocking agents and yielded inconsistent conclusions regarding its role in these diseases. CLCA1 not only regulates mucin expression, but also participates in innate immune responses by binding to yet unidentified molecules on inflammatory cells for cytokine and chemokine production. CLCA1 also targets lymphatic endothelial cells and cancer cells by regulating lymphatic cell proliferation and lymphatic sinus growth in the lymphatic organs and controlling cancer cell differentiation, proliferation, and apoptosis, all which depend on the location of the lymphatic vessels, the type of cancers, the presence of Th2 cytokines, and possibly the availability and type of CLCA1-binding proteins. Here we summarize available studies related to these different activities of CLCA1 to assist our understanding of how this secreted modifier of calcium-activated chloride channels (CaCCs) affects mucus production and innate immunity during the pathogenesis of respiratory, gastrointestinal, and malignant diseases.

## Introduction

The calcium-activated chloride channel regulator (CLCA, previously known as chloride channel calcium activated) proteins^1^ are a family of secreted self-cleaving, and zinc-dependent metalloproteases that activate calcium-dependent chloride currents in mammalian cells.^2^^,^^3^ CLCA family members are abundantly expressed in mucosal epithelia and regulate both chloride transport and mucin expression.^2^ They serve as a secreted modifier of calcium-activated chloride channels (CaCCs) as well as cell adhesion molecules.^4^, ^5^, ^6^, ^7^ Several CLCA members have been identified from different species, including human, mouse, rat, cow, pig, horse, and dog.^1^^,^^6^^,^^8^, ^9^, ^10^, ^11^, ^12^, ^13^ For example, four human (hCLCA1 to hCLCA4), eight murine (mCLCA1 to mCLCA8), and four bovine (bCLCA1, bCLCA2 (Lu-ECAM-1), bCLCA3, and bCLCA4) CLCA family members have been reported.^14^ Yet, the tissue expression patterns of hCLCA1, mCLCA1, and bCLCA1 can be different. hCLCA1 is expressed in the intestinal epithelia,^6^ mCLCA1 in multiple tissues,^9^ and bCLCA1 in the respiratory epithelia of trachea and bronchi.^1^ Indeed, the hCLCA1 ortholog in mice is not mCLCA1, but mCLCA3 and also called goblet cell protein-5 (Gob-5) with a similar expression profile to that of hCLCA1.^15^ Therefore, the nomenclature of this family of proteins has been confusing. To be identiﬁed among the species, mouse mCLCA3 (Gob-5) is now renamed as mCLCA1 according to the Human Gene Nomenclature Committee and the Rat Genome Database.^16^ Therefore, we use mCLCA1 instead of mCLCA3 or Gob-5 in this review. Despite in mucus-producing diseases, there is scarce information available regarding the pathobiological functions of CLCA1 in humans and animals. This review summarizes the limited functional studies of CLCA1 in respiratory diseases, cancers, and gastrointestinal diseases from humans and experimental models to promote the investigation of this molecule.

## Characterization of CLCA1 in the CLCA family

Human hCLCA1 is the first member to be investigated in the family of Ca^2+^-activated Cl^−^ channel regulators. Its genomic sequence comprises 31,902 base pairs with 15 exons and 14 introns located on the short arm of chromosome 1 (p22–31).^6^ Northern blot analysis showed hCLCA1 expression mainly in small and large intestine and colon mucosa.^6^ In mice, mCLCA1 exhibits a similar expression profile to that of hCLCA1. The murine mCLCA1 gene is located on the respective syntenic locus of chromosome 3 (band H2–H3),^17^ and it is expressed in secretory epithelial cells, squamous epithelia, and a subset of lymphocytes.^6^

CLCA1 appears to participate in chloride conductance across the epithelial cell membrane and therefore affects epithelial fluid secretion, mucous production, cell signaling, cell adhesion, cell cycle control, apoptosis, tumorgenesis, and metastasis.^18^ Previous investigations have challenged its character as being a Ca^2+^-dependent Cl^−^ channel itself, largely because of the lack of appropriate membrane-spanning domains based on the algorithm predictions, the absence of attachment to the cell membrane, its localization to the mucin granules during secretion into the extracellular milieu as a globular protein, and functional analysis in nonheterologous cell lines.^15^^,^^19^, ^20^, ^21^ Moreover, CLCA1 elevates the conductance of endogenous Ca^2+^-dependent Cl^−^ channels by lowering the energy barriers for ion translocation through the pore instead of forming Ca^2+^-dependent Cl^−^ channels *per se*.^22^

Biochemical and electrophysiological studies demonstrate that hCLCA1 and mCLCA1 are secreted as globular proteins without trans-membrane domains, and they could be removed from the cell surface and identified in the extracellular milieu when expressed in the epithelial cell line HEK293 cells.^23^ They may participate in the extracellular signaling and protein-protein interactions depending on the presence of the von Willebrand factor type A (VWA) and fibronectin type III domains.^21^^,^^23^ Therefore, CLCA1 may act indirectly as a regulator of CaCCs through the identified ion channels,^4^^,^^5^ rather than as a genuine chloride channel protein.^23^ Co-expression of the β-subunit KCNMB1 (potassium calcium-activated channel subfamily M regulatory beta subunit 1) with CLCA1 evoked significantly the CaCCs in human embryonic kidney cells, suggesting that the activation of CLCA1 can be adjusted by accessory subunits.^24^ Prior studies reported a self-cleavage of the 110-kDa full-length mCLCA1 post-translational product in the endoplasmic reticulum into a 75~90-kDa NH_2_-terminal and a 35-kDa COOH-terminal proteins that underwent glycosylation,^21^ and they demonstrated that the NH_2_-terminal fragment was essential and sufficient to activate CaCCs in HEK293T cells.^2^ Transmembrane protein 16A (TMEM16A, also known as anoctamin-1 [ANO1] or discovered on gastrointestinal stromal tumors-1 [DOG1]) was described as the first genuine CaCC in mammals.^25^, ^26^, ^27^ Secreted CLCA1 regulates the TMEM16A-dependent calcium-activated chloride currents, and this activation occurs in a paracrine fashion.^5^ Exogenous soluble CLCA1 co-localizes with and improves the function of TMEM16A on cell surface.^5^ The VWA domain within the cleaved CLCA1 NH_2_-terminal fragment is essential and sufficient for this interaction.^4^

## Roles of CLCA1 in respiratory diseases

**Fig. 1:**
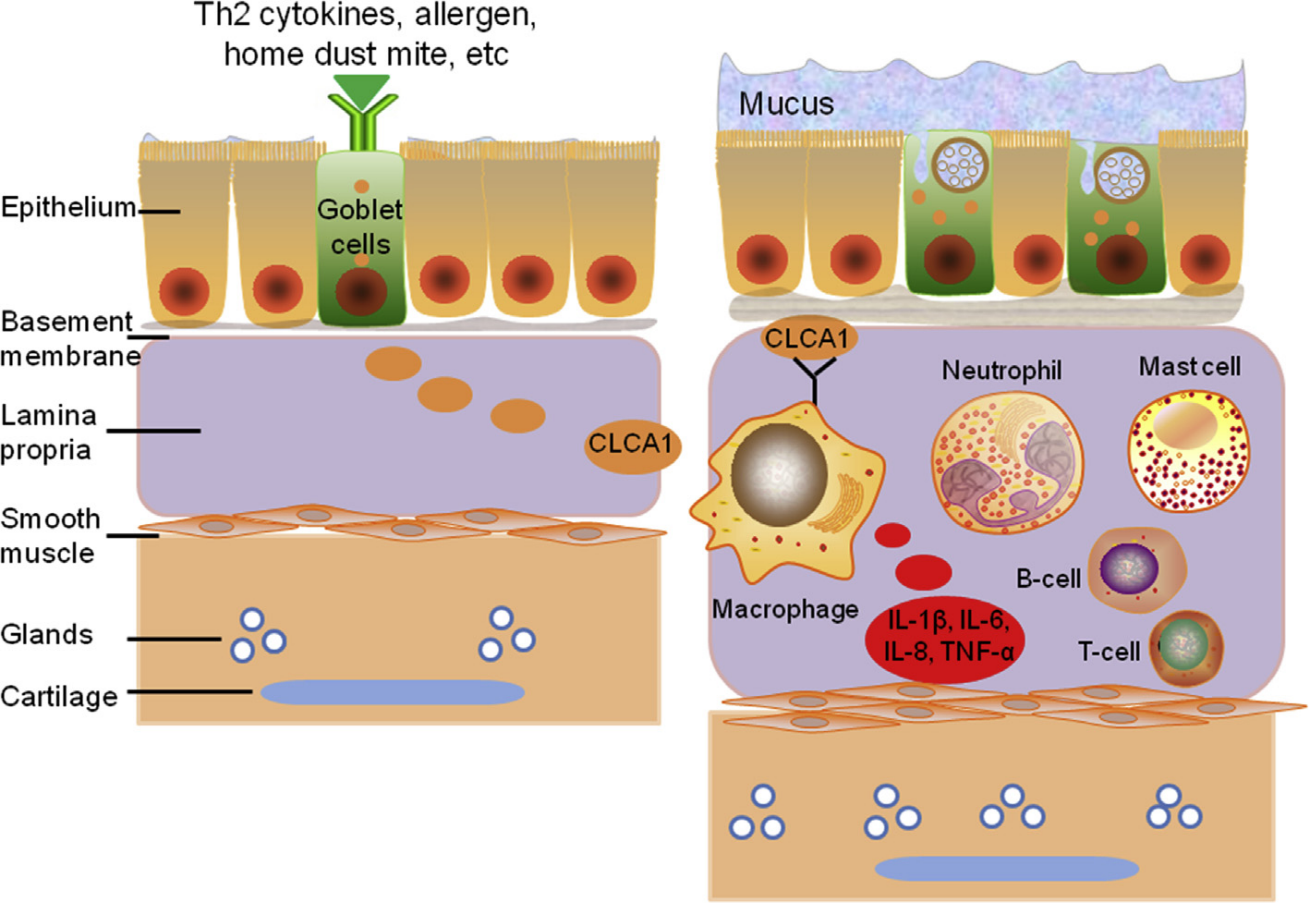
CLCA1 in the respiratory system. Expression of CLCA1 is induced in the lung epithelium by T helper 2 (Th2) cytokines, allergen, or house dust mite (HDM) treatment and causes increased mucus production. In addition to mucus secretion modulation, CLCA1 is also an essential regulator of innate immune responses. The secreted form of CLCA1 can act as a signaling ligand that activates monocytes and alveolar macrophages in a dose-dependent manner, increase the levels of pro-inflammatory cytokines and chemokines (IL-1β, IL-6, IL-8 and TNF-α), and initiate the recruitment of inflammatory cells, such as B cells, T cells, neutrophils, and mast cells

CLCA1 is probably the best-studied member from the CLCA family prominently in human respiratory diseases. Human hCLCA1 is a crucial mediator of hypersecretory lung diseases, such as asthma,^28^ chronic obstructive pulmonary disease (COPD),^29^ cystic fibrosis, and other diseases that manifest increased mucus production. In addition to the modulation of airway mucus secretion, CLCA1 is also involved in the modulation of tissue inflammation in the innate immune response by regulating the production of cytokines and chemokines (Fig. 1). The secreted form of CLCA1 acts as a signaling ligand that activates human monocyte U-937 and primary cultured porcine alveolar macrophages in a dose-dependent manner and increases the expressions of pro-inflammatory interleukin (IL)-1β, IL-6, IL-8 and tumor necrosis factor-α (TNF-α), thereby acting as a pleiotropic factor in lung inflammation.^30^ CLCA1-induced chemokines may initiate the recruitment of inflammatory cells to the respiratory epithelium and lamina propria (Fig. 1). Accordingly, hCLCA1 and its ortholog mCLCA1 in mice have been proposed as a biomarker of inflammatory airway diseases (IAD). These novel biomarkers may help potentiate the treatments of patients with or at high risk of IAD by targeting these human or mouse CLCA1.

### Asthma

Goblet cell hyperplasia and mucus overproduction are among the main pathological events that characterize asthma.^22^ A signiﬁcant correlation between mucus overproduction and increased morbidity and mortality was found in asthmatic patients.^31^ Studies have shown that overexpression of hCLCA-1 and the murine mCLCA-1 is essential to mucin overproduction and airway hyperresponsiveness in asthma. hCLCA1 was identified as one of the up-regulated genes with the highest log2 fold change (LFC) (LFC = 2.81) in an integrative analysis of multiple public microarray data sets that aimed to determine differentially expressed genes in asthmatic subjects.^32^ hCLCA1 was upregulated in the epithelium in mild asthma and suppressed by corticosteroids.^33^ hCLCA1 expression marked IL-13 centric Th2-driven inflammation in asthmatic patient nasal airway.^34^ Expression of mCLCA1 was specifically up-regulated in the lung epithelium of IL-9 transgenic mice as well as by intratracheal instillation of IL-9 and other Th2 cytokines (IL-4, IL-13); and the expression of hCLCA1 was also promoted by Th2 cytokine treatment in cultured human primary lung cells, suggesting the participation of hCLCA1 and mCLCA1 in the pathogenesis of Th2 cytokine-mediated asthmatic disorders in humans and experimental models.^35^

The function of CLCA1 is not restricted to Th2 stimuli. Using a house dust mite allergen (HDMA)-induced experimental asthma in cynomolgus macaques, CLCA1 was up-regulated in the bronchoalveolar lavage fluid (BALF) as determined by mass spectrometry; and significantly reduced in the same challenged but corticosteroid-treated animals.^36^ Repeated intranasal administration of cerium dioxide nanoparticles (CeO_2_NPs) in the presence of HDM initiated a type 2 immune response in mice, which presented within asthmatic endotypes and accompanied by increases of mucin and inflammatory regulator mCLCA1.^37^ By examining the gene expression patterns in ovalbumin (OVA)-sensitized and -challenged male Balb/c mice, mCLCA1 mRNA was induced specifically in the murine lung tissue with airway hyperresponsiveness.^38^ mCLCA1 gene expression silencing with the adenovirus expressing mCLCA1 antisense RNA inhibited the bronchial hyperreactivity and mucus hypersecretion. Meanwhile, overexpression of the mCLCA1 gene by administrating adenovirus containing the mCLCA1 gene intratracheally aggravated the murine asthma phenotype, including eosinophil infiltration, goblet cell metaplasia, excessive mucus production, and bronchial hyperreactivity.^38^ OVA-challenged mCLCA1-deficient *Clca1*^*−/−*^ mice showed decreased peri-vascular tissue inflammation, goblet cell hyperplasia, mucus production, as well as decreased airway hyperresponsiveness after cholinergic provocation with methacholine.^39^ mCLCA1 antibody treatment remarkably reduced airway inflammation and goblet cell numbers in lung tissue, and promoted goblet cell apoptosis with increased production of Bax and decreased expression of Bcl-2 in goblet cells. mCLCA1 antibody significantly reduced the production of mucin 5AC (MUC5AC, the major respiratory mucin in goblet-cell secretion^40^) and IL-13 in BALF.^41^ Collectively, mCLCA1 presents an essential activity in murine asthma, and its human counterpart hCLCA1 may become an effective therapeutic target for asthma.

Yet, the allergic response produced by acute intranasal IL-13 instillation or OVA challenge was similar in *Clca1*^*−/−*^ mice and their wild-type (WT) littermates from a different but similar study, which demonstrated that the expression of mCLCA1 is not required for mucin hypersecretion regulated by pro-inflammatory signals in mice.^42^ Similar to the observations from *Clca1*^*−/−*^ mice, siRNA transfection-mediated knockdown of the hCLCA1 gene expression in human lung epithelial cell line (NCI–H292) failed to reduce the MUC5AC mRNA level or protein production.^42^ Contradictory observations from these different studies have made the roles of CLCA1 in mucus overproduction and asthmatic responses inconclusive.

### Chronic obstructive pulmonary disease

**Fig. 2:**
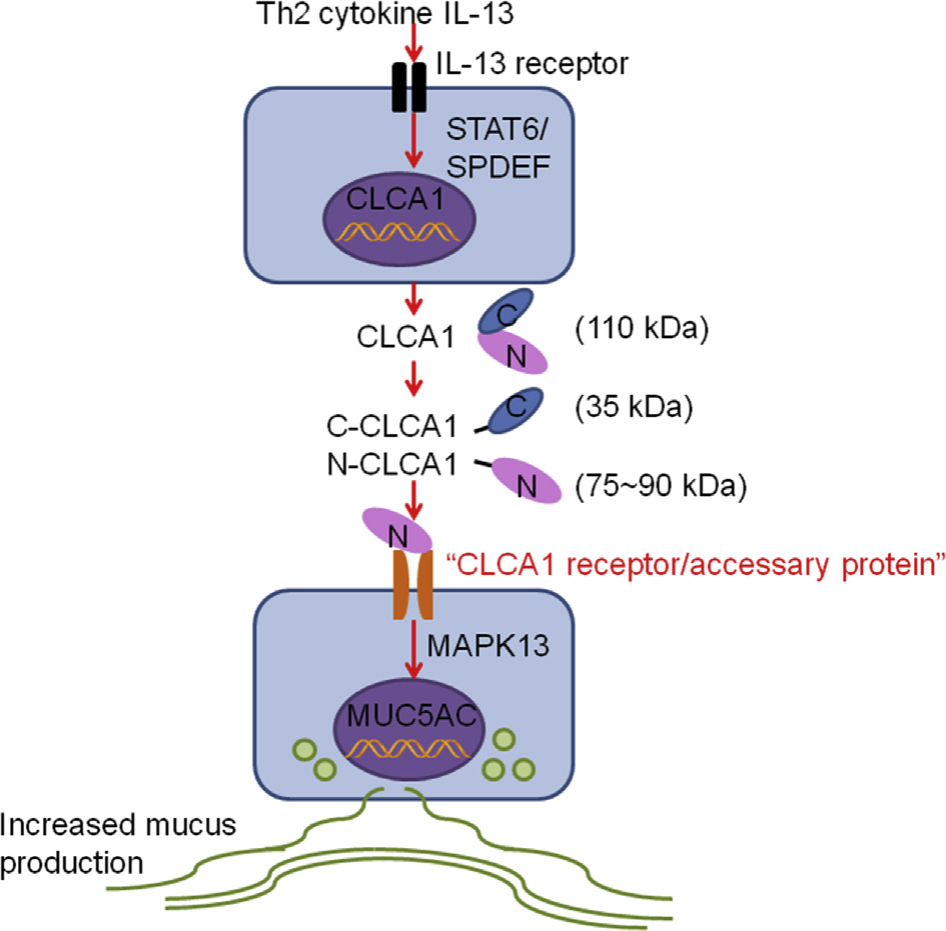
CLCA1 on mucus production. Th2 cytokine IL-13 binds to the IL-13 receptor and leads to increased expression of CLCA1 via the STAT6/SPDEF signaling/transcription pathway. The 110-kDa full-length CLCA1 translational product in the endoplasmic reticulum undergoes self-cleavage by releasing the 35-kDa COOH-terminal fragment and allowing the 75~90-kDa NH_2_-terminal protein to bind to a putative yet undefined receptor on the airway epithelial cell surface, followed by activation of the MAPK13 signaling pathway and mucin gene expression

COPD is a chronic inflammatory lung disease with features of goblet cell hyperplasia and mucus overproduction. Twenty-two novel single nucleotide polymorphisms (SNPs) of the hCLCA1 gene were identified in COPD subjects from Japanese and Egyptian populations that might be effective for anticipating the susceptibility to COPD.^29^ Real-time quantitative PCR analyses revealed higher hCLCA1 mRNA level in hypertonic saline-stimulated sputum cells from COPD patients, compared with those of non-smoker controls (*P* = 0.02). Immunostaining with an anti-hCLCA1 antibody showed prominent hCLCA1 production in the bronchioles and terminal bronchioles epithelia in COPD patients.^43^ A significant increase of hCLCA1 and MUC5AC mRNA and their protein expression was found in patients with COPD compared with those without COPD.^44^ hCLCA1 level associated with MUC5AC expression across the lung samples from patients with or without COPD.^44^ Excessive mucus production due to COPD may be resulted from the signaling pathway from hCLCA1 to MAPK13 (Fig. 2).^44^ Therefore, targeting hCLCA1 might have potential clinical benefits to COPD patients, although a direct role of CLCA1 in COPD has not been tested.

### Cystic fibrosis

Cystic fibrosis (CF) is a frequent autosomal recessive disorder induced by mutations in the cystic fibrosis transmembrane conductance regulator (CFTR) gene, which encodes a cyclic adenosine monophosphate (cAMP)-dependent chloride channel.^45^ CaCCs contribute to airway Cl^−^ and fluid secretion, thereby modulating disease severity.^46^ mCLCA1 has been proposed to mediate the CF-modulatory role of CaCCs.^46^ The tissue expression pattern of mCLCA1 overlaps with that of CFTR, indicating that both genes participate in the pathogenesis of CF.^9^

Members of the CLCA gene family may act as modulators of the CF phenotype in affected patients and murine experimental CF.^47^^,^^48^ The percentages of hCLCA1-positive epithelial cells (nasal polyps, 93.8% ± 7.2%; nasal mucosa, 85.4% ± 12.3%; sinus mucosa, 71.4% ± 18.7%) were significantly higher in the epithelium of patients with CF compared with that of control subjects (20.8% ± 15.1%; *P* < 0.05).^49^ Furthermore, hCLCA1 protein is profoundly up-regulated in the bronchial mucosa of patients with CF.^50^ Stimulation with Th2 cytokines IL-4, IL-9, and IL-13 significantly increased the hCLCA1 protein expression (*P* < 0.05) in mucosal tissue explant from the CF patient upper airways, while MUC5AC mRNA and mucin protein levels were not significantly altered, which questioned the role of hCLCA1 as a mediator of mucus hypersecretion in CF.^51^ Similarly, mouse mCLCA1 is expressed in the respiratory goblet cells and has been linked to the secretory dysfunction in experimental CF.^15^ The mCLCA1 mRNA is readily detected by northern blot analysis and by *in situ* hybridization in the respiratory epithelia of trachea and bronchi as well as epithelia of their submucosal glands.^8^ The similarity between the tissue expression patterns of mCLCA1 and CFTR underscores the potential importance of CLCA1 in CF.^8^ However, whether the secreted CLCA1 proteins interact directly or indirectly with either the CFTR protein or others as yet unidentified channel protein *via* a receptor-mediated pathway remains unknown.^52^ Therefore, the mechanism of suspected modulatory functions of CLCA1 in CF is far from being resolved.

Prior studies showed that the mRNA level of mCLCA1 is not differentially regulated in the respiratory tract of murine experimental CF compared with WT controls.^53^ Basal bioelectric measurements failed to reveal any significant differences in basal short-circuit current, amiloride-sensitive Na^+^ absorption, cAMP-dependent Cl^−^ secretion, and activation of Ca^2+^-activated (uridine-5′-triphosphate-mediated) Cl^−^ secretion in *Clca1*^*−/−*^ mice compared with WT mice.^46^ Intratracheal administration of IL-13 generated an approximately 30-fold up-regulation of the mCLCA1 transcripts without inducing the CaCCs activity in WT mouse airways, and induced goblet cell hyperplasia and mucin gene expression to the similar levels in both genotypes.^46^ Reverse-transcription quantitative PCR assay did not detect significant changes in the expression of other CaCC candidates that may compensate for a lack of mCLCA1 function, including seven mCLCAs, mBEST1, mBEST2, mCLC4, mTMEM16A, and mTTYH3, in the lung between *Clca1*^*−/−*^ and WT mice.^46^ These findings argue against the function of mCLCA1 in mediating its CF-modulatory role through CaCC conductances in murine respiratory epithelia. Therefore, like in asthma, the role of mCLCA1 in CF also remains inconclusive.

### Pneumonia

Bacterial *Staphylococcus aureus* (*S. aureus*) infection induces pneumonia. Female *Clca1*^*−/−*^ mice that were transnasally inoculated with *S. aureus* showed decreased neutrophil recruitment to the bronchoalveolar space and decreased mRNA and protein levels of IL-17 and murine C-X-C motif chemokine ligand 1 (CXCL-1) compared to those from infected WT controls. It was suggested that mCLCA1 modulated the leukocyte accumulation *via* induction of IL-17 and CXCL-1 in bacterial pneumonia and appeared to have an effect on the early innate immune response following *S. aureus* lung infection.^54^ Yet, mCLCA1-deficiency did not affect mucus cell number and mucin secretion in infected mice. In murine experimental acute pneumonia induced by *S. aureus* infection, the expression of airway mucus component bactericidal/permeability-increasing protein (BPI) fold-containing family A member 1 (Bpifa1), a secretory protein from the respiratory tract that has antimicrobial and anti-biofilm properties to regulate mucociliary clearance,^55^ was significantly intensified in *Clca1*^*−/−*^ mice compared to that from WT mice at 24 hours post-infection.^56^ Therefore, the role of CLCA1 in respiratory diseases might involve much more complicated downstream pathways, rather than just goblet cell mucus production and epithelial cell chloride ion secretion.

### Mucin synthesis

Mucus overproduction contributes to airway inflammation and obstruction. Previous studies showed that Th2 cytokine IL-13 receptor activation led to the activation of the IL-13 signaling molecule STAT6 (signal transducer and activator of transcription 6)^57^ and consequent induction of CLCA1 gene expression, and associated with the secretion of MUC5AC in airway epithelial cells. Specific knockdown of transcription factor SPDEF (sterile alpha motif [SAM] domain-containing prostate-derived Ets transcription factor) suppressed IL-13-induced MUC5AC production in human airway epithelial cells. Repression of STAT6 prohibited the production of IL-13-induced SPDEF and MUC5AC. Therefore, IL-13 induces MUC5AC production *via* the STAT6/SPDEF signaling and transcription pathway in human airway epithelial cells (Fig. 2).^58^ Expression of CLCA1 was up-regulated by IL-13 but down-regulated by SPDEF siRNA,^58^ proposing a possibility of preventing mucus overproduction in chronic airway inflammatory diseases by targeting the STAT6/SPDEF signaling and transcription pathway.

#### Cigarette smoke-induced mucin production

Immunostaining of airway epithelium with an anti-hCLCA1 antibody demonstrated significant enhancement of hCLCA1 expressin in smokers without COPD (*P* = 0.02) compared with that in non-smokers.^43^ Cigarette smoke-exposed Sprague-Dawley rats showed significant up-regulation of rat CLCA1, epidermal growth factor receptor (EGFR), and MUC5AC in the trachea and lung tissues as well as the numbers of goblet cells in the tracheal epithelium from day 21 onwards than those from the non-smoking group (*P* < 0.001).^59^ Niflumic acid (NFA, blocker of the CLCAs), AG-1478 (inhibitor of EGFR tyrosine kinase), or combination of the two inhibited the smoke-induced MUC5AC gene expression.^59^ In human bronchial epithelial cell line NCI–H292, cigarette smoke solution up-regulated the expression of hCLCA1, EGFR, and MUC5AC.^59^ Cigarette smoke markedly influenced the IL-13-induced Th2-signature gene expression and reduced hCLCA1 and MUC5AC expression in air-liquid interface differentiated human bronchial (ALI-PBEC) and tracheal epithelial cells (ALI-PTEC).^60^ Both *in vivo* and *in vitro* studies demonstrated an essential role for CLCA1 in cigarette smoke-induced mucin synthesis. The activity of cigarette smoke in promoting mucin expression may vary depending on the presence of Th2 cytokines.

#### TNF-α–induced mucin expression

In an *ex vivo* model of human upper airway mucosa, stimulation with TNF-α (10 ng/mL) for 24 hours also significantly increased the hCLCA1 and MUC5AC mRNA levels as well as hCLCA1 and mucus protein levels.^61^ Blocking hCLCA1 functions with chloride channel blockers (NFA and MSI-2216) reduced the MUC5AC mRNA levels and mucus protein expression in a dose-dependent manner.^61^ This study suggests that inhibition of hCLCA1 is potentially a novel approach to inhibit mucus expression. Specific blockers to target hCLCA1 may have significant clinical value and merit further investigation.

## Roles of CLCA1 in cancer progression

Results from previous studies have documented close association of CLCA1 with cancer progression.^62^, ^63^, ^64^ Expression of hCLCA1 antagonizes the survival of mammary MCF7 tumor cells by sensitizing them to anoikis (detachment-induced apoptosis).^65^ Reduced expression of hCLCA1 predicts disease relapse and poor survival in patients with colorectal cancer,^64^ and is an independent risk factor of poor disease-free survival in patients with pancreatic ductal adenocarcinoma.^66^ A genetic study using fixed colorectal carcinoma samples also indicated a negative association between hCLCA1 level and the cancer stage. Each colorectal carcinoma stage increase reduced the hCLCA1 by 3.1 folds.^67^ Yet, evidence from ovarian cancer analysis revealed that CLCA1 was overexpressed during cancer progression, as determined by both RT-PCR and immunoblot analyses. hCLCA1 silence with siRNA blocked the ovarian cancer cells multicellular aggregates.^68^ Therefore, the role of CLCA1 in tumor progression may depend on the types of tumors. Further understanding of how CLCA1 exerts its function in different metastatic progression patterns across major human cancers and how CLCA1 affects cancer cell resistance to chemotherapeutic drugs may lead the identification of potential therapeutic targets and development of effective approaches to treat these relevant cancers.

### CLCA1 in ovarian cancer

Comparative proteomics analysis revealed an increased expression of hCLCA1 in an ovarian cancer cell line OV-90 and cell models of tumor aggregate formation (TOV-112D and ES-2).^68^ During the process of ovarian cancer metastasis, detached cancerous cells tend to form multicellular aggregates that facilitate ovarian cancer metastasis both by breaching the mesothelium and initiating widespread peritoneal dissemination.^69^ It was shown that knockdown of hCLCA1 with siRNA reduced the ability of cancer cells to form multicellular aggregates.^68^ These findings indicate that hCLCA1 plays an essential role in promoting ovarian cancer metastasis, although further experimental validation is required to characterize the function of CLCA1 in the pathogenesis of ovarian cancer.

### CLCA1 in human colorectal cancer

Different from what was reported in the ovarian cancer cells, a proteogenomic study applying mass spectrometry and gene microarray in both human colorectal cancer and adjacent normal mucosa tissues confirmed reduced expression of hCLCA1 in the colorectal cancer tissue.^70^ Serum concentration of hCLCA1 was also significantly lower in colorectal cancer patients than that from healthy controls (*P* < 0.01),^71^ indicating that hCLCA1 may serve as a potential biomarker of human colorectal cancer. Low levels of hCLCA1 expression in the colonic epithelial tissues correlated with a less differentiated tumor histological grade, an advanced tumor stage, metastases in regional lymph nodes, and high Dukes stage that has been used to grade colon cancer based on cancer cell locations from the mucosa, muscle layer, to lymph node, and distant metastasis. Patients with low hCLCA1 expression levels had significantly poorer overall survival rate and higher recurrence rate than those with high levels of hCLCA1 expression (*P* < 0.05).^64^ Results from a clearly-defined cohort of patients with rectal cancer who received concurrent chemoradiotherapy before tumor resection surgery implied that high levels of hCLCA1 associated significantly with higher pre-treatment tumor nodal stages, inferior tumor regression grade, and vascular invasion.^72^ High immunohistochemical expression of hCLCA1 also predicted shorter survivals in patients with rectal cancer who received concurrent chemoradiotherapy before surgery.^72^ hCLCA1 expression is up-regulated in sodium butyrate-differentiated human intestinal cancer cell line Caco-2. hCLCA1 knockdown with siRNA inhibited Caco-2 cell differentiation but promoted cell proliferation. Therefore, hCLCA1 may serve as a useful diagnostic biomarker for colorectal cancer prognosis.^63^ Loss of CLCA1 expression prevents enterocyte differentiation and may promote colonic cancer progression. Up-regulation of hCLCA1 suppressed colorectal cancer progression and metastasis *in vivo* and *in vitro*, whereas blocking hCLCA1 expression led to the opposite phenotypes.^71^ Elevated expression levels of hCLCA1 repressed the Wnt/β-catenin signaling as well as the epithelial-mesenchymal transition (EMT) process in colorectal cancer cells, suggesting that hCLCA1 acted as a tumor suppressor in colorectal cancer by suppressing the Wnt/β-catenin signaling pathway and EMT process.^71^ Indeed, immunohistochemical analysis of human cancerous colonic epithelial tissues reveals positive correlation between hCLCA1 staining with E-cadherin and tumor suppressor p53.^64^ All these studies support both the therapeutic and prognostic potential of hCLCA1 in rectal cancers, although the mechanisms by which hCLCA1 suppresses tumor growth may beyond inhibiting the Wnt/β-catenin signaling network and EMT process.

### CLCA1 in lung cancer

B16–F10 melanoma cells are characterized by robust surface expression of β_4_ integrin^73^ and consistent high lung colonization potential. Previous studies demonstrated that glutathione S-transferase fusion proteins of β_4_-binding motifs (β_4_BMs) from the 90- and 35-kDa mCLCA1 subunits bound to the β_4_ integrin in a metal ion-dependent manner.^74^ The endothelial mCLCA1 serves as the adhesion receptor for the β_4_ integrin expressed at high levels on lung metastatic B16–F10 cells.^75^ The β_4_ integrin-mCLCA1 interaction led to complex formation with and activation of the downstream focal adhesion kinase (FAK)/extracellular signal-regulated kinase (ERK) signaling, and promoted early, intravascular, and metastatic growth.^75^ Different from aforesaid in colorectal cancer, however, mCLCA1 in lung cancer appears to play a positive role by promoting cancer cell migration and metastasis after binding to the β_4_ integrin.

## CLCA1 in gastrointestinal diseases

In the gastrointestinal tract, CLCA1 is expressed predominantly in the small intestine, colon, and appendix. As a goblet cell secretory protein from the respiratory tract, CLCA1 can also present as a secretory protein and perform important functions in intestinal mucus dynamics and homeostasis.^76^^,^^77^ A recent investigation indicated that the endogenous metallohydrolase activity of CLCA1 is required for intestinal mucus processing.^76^ The localization of mCLCA1 protein in salivary duct cells, parietal cells, and epithelial cells of the small intestinal crypts as well as on the pancreatic zymogen granule membrane favors a cell type-specific function of mCLCA1 contributing to transepithelial ion transport and protein secretion.^78^ Further understanding of CLCA1 function in mucus production helps probe the pathophysiology of gastrointestinal diseases and develop novel treatment for mucus production-associated complications by regulating CLCA1 expression.

### CLCA1 in murine dextran sodium sulfate-induced colitis

To analyze the role of CLCA1 in the pathogenesis of murine dextran sodium sulfate (DSS)-induced colitis, mCLCA1-deficient *Clca1*^*−/−*^ mice (C57BL/6J background) and WT (same genetic background) mice under unchallenged or DSS-challenged conditions at different time points were compared. Lack of mCLCA1 correlated with a more than two-fold increase of IL-17 and CXCL-1 mRNA in the distal colon during the development of DSS-induced colitis, similar to what was reported from *S. aureus-*induced pneumonia, although accumulation of neutrophils, macrophages, and lymphocytes in the proximal and distal colon following colitis induction did not differ between *Clca1*^*−/−*^ and WT mice. Further, no difference was found between *Clca1*^*−/−*^ and WT mice under unchallenged or DSS-challenged conditions in terms of clinical parameters, disease progression or outcome, and key histopathological parameters such as epithelial defects and regeneration.^79^ Similarly, when comparing *Clca1*^*−/−*^ and WT mice under naive and at different time points of DSS-challenged conditions, expression of the major intestinal mucin MUC2 and mucus structure, or other essential components of the mucus including Agr2, Fcgbp, Klk1 and Zg16, or mucus growth rate, thickness, and penetrability also did not differ. DSS challenge identically affected the mucus barriers, bacterial translocation, fecal microbiota composition, fecal blood content and stool consistency scores of *Clca1*^*−/−*^ and WT mice, suggesting that CLCA1 is not required for mucus synthesis, structure and barrier function in murine colon.^16^ This discrepancy with the results of CLCA1 function in the lung highlights the different effects of the type of challenges and tissue environment on the roles of CLCA1.

### CLCA1 in cystic fibrosis intestinal mucous disease

CLCA1 is important for the function of intestinal goblet cell, including adjustment of the mucous properties or secretion that is changed in CF intestinal disease. A significant correlation between allelic variants at the human hCLCA1 gene locus and CFTR-independent chloride conductance was found in rectal mucosa biopsies from CF subjects.^80^ Mucin secretion in the CF colon depended on the CFTR expression and associated with the production of mCLCA1.^81^ It was shown that the expression of mCLCA1 was significantly down-regulated in small intestines from congenic C57BL/6 CF mice.^82^ In TgrIFABP-mCLCA1 transgenic mice, where mCLCA1 expression was driven by the rat intestinal fatty acid binding protein promoter (rIFABP) that is specific to intestinal enterocytes and goblet cells,^83^ up-regulation of mCLCA1 resulted in significant improvement of the mucous-based intestinal lesions and disease amelioration.^82^ Goblet cells were significantly larger in the intestine of TgrIFABP-mCLCA1 CF mice than WT CF mice, and the number of goblet cells per villus in the TgrIFABP-mCLCA1 CF mouse ileum was significantly elevated over the control mice with CF alone, supporting a role of mCLCA1 in goblet cell hypertrophy and hyperplasia. Together, these results suggest that mCLCA1 also displays activities in controlling mucous properties and secretion during the pathogenesis of intestinal mucous diseases.^82^ Secreted (extracellular) mCLCA1 may inhibit goblet cell degranulation as a negative feedback mechanism to control the expression of extracellular mucus.

### CLCA1 in ulcerative colitis

Ulcerative colitis is an inflammatory disease of the colonic mucosa caused by a sustained deregulation of the intestinal epithelial barrier function.^84^ Whole genome expression analysis revealed that a group of genes was differentially regulated in the epithelial organoid cultures (EpOCs) and differentiated EpOCs (d-EpOCs) in patients with ulcerative colitis,^84^ including a marked down-regulation of hCLCA1, which is a major component of the mucus produced mainly by secreting cells of the distal digestive tract.^77^ A loss of hCLCA1 protein expression was also sporadically detected in the colonic epithelium of whole intestinal samples from subjects with ulcerative colitis.^84^ These results provide evidence of a lasting epithelial defect in mucus barrier quality that correlates with the progression of ulcerative colitis.^85^

### CLCA1 in parasitic nematode infection

Acidic mammalian chitinase (AMCase) is a major digestive enzyme that constitutively degrades chitin substrates and produces (GlcNAc)_2_ fragments in mouse gastrointestinal environment. AMCase is known to be induced by allergens and helminthes. AMCase-deficient mice showed a profound deficiency in type 2 immunity following infection with the chitin-containing gastrointestinal nematodes *Nippostrongylus brasiliensis* and *Heligmosomoides polygyrus bakeri*. This impaired immunity was correlated with attenuated mucus synthesis and reduced intestinal expression of the signature type 2 immune response genes *Chil3, Clca1*, *Il13,* and *Retnlb,* indicating that AMCase acts as a crucial initiator of protective type 2 immune responses to intestinal nematode infection in the gastrointestinal tract.^86^ The mucous cell-associated CLCA1 transcript was also one of the most up-regulated transcripts observed from immunized sheep in response to infection from ovine gastric nematode *Teladorsagia circumcincta*.^87^ These studies also support an essential role of CLCA1 in regulating mucus production in mammalian responses to parasitic nematode infection.^87^

## CLCA1 in the lymphatic system

**Fig. 3:**
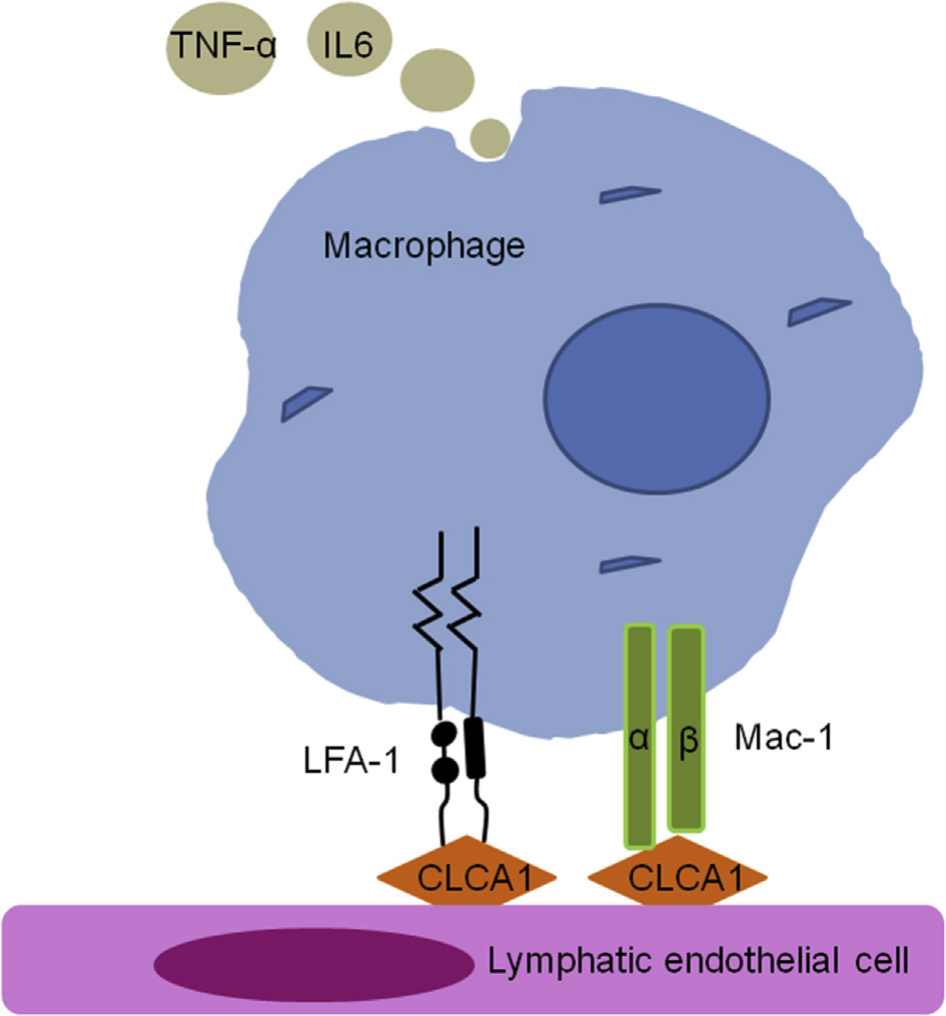
CLCA1 from the lymphatic endothelium. CLCA1 can serve as a lymphatic endothelial surface ligand by binding to the β2 integrins LFA-1 and Mac-1 that are preferentially expressed on macrophages, and play a role between lymphatic cell and immune cell interactions

Lymph nodes undergo extensive architectural remodeling during immune responses. It involves expansion of the lymphatic sinuses (lymphangiogenesis) accompanied by up-regulated stromal components, including lymphatic endothelial cells (ECs), blood ECs, fibroblast reticular cells, and CD4^–^CD8^–^ double negative T cells.^88^^,^^89^ Lymph node remodeling is involved in immune responses and in many inflammatory diseases such as cancer and arthritis.^89^ A hamster monoclonal antibody 10.1.1 recognized lymphatic endothelial mCLCA1. This antibody identiﬁed mCLCA1 as a 90-kDa cell-surface protein from lymphatic endothelium and stromal cells within mouse spleen and thymus. mCLCA1 serves as a lymphatic endothelial surface ligand by binding to the β2-integrins LFA-1 (lymphocyte function-associated antigen 1) and Mac-1 that are preferentially expressed on granulocytes, NK cells, dendritic cells, and macrophages, and mediate the adhesion of Mac-1- or LFA-1-positive leukocytes to lymphatic ECs and lymph node lymphatic sinuses (Fig. 3).^90^ Expression of mCLCA1 on lymphatic ECs was not affected by TNF-α/IL-1β stimulation, indicating that mCLCA1 is a constitutive biomarker of normal and inflamed lymphatic ECs.^90^ Antibody 10.1.1 induction of lymph node lymphatic EC proliferation and lymphatic sinus growth appears to be a specific consequence of antibody binding to mCLCA1 on lymph node lymphatic ECs. Antibody 10.1.1-induced lymphangiogenesis was restricted to lymph nodes, as mCLCA1-expressing lymphatic vessels of the jejunum and dermis were unaffected at 23 h after antibody 10.1.1 treatment.^89^ Taken together, these results established a crucial function of mCLCA1 in coordinating lymph node remodeling during immune responses and suggest a new strategy for a therapeutic manipulation of the immune responses.

## Conclusion

CLCA1 as a regulator of Ca^2+^-activated chloride ion transport on epithelial cells, mucin expression from goblet cells, cytokine and chemokine expression from monocytes and macrophages, tumor cell migration and metastasis, and proliferation of lymphatic ECs, it has been tested in respiratory diseases, gastrointestinal diseases, cancers, and lymphatic tissue remodeling in humans and experimental models (Table 1). Some confusing but potentially important observations are that the same CLCA1 acted differently from different types of cancers and the similar asthma studies yielded contradictory observations. Although limited amounts of work have been carried to test a direct role of CLCA1 in these diseases using the mCLCA1-deficient *Clca1*^*−/−*^ mice, observations from conventional biochemical and immunological studies proposed several possible mechanisms by which CLCA1 participate in these diseases. The pathobiological activities of CLCA1 may vary depending on the environment of different types of diseases, the presence of type 2 cytokines, and very interestingly the types of integrins or other accessary proteins. CLCA1 was initially identified as a Ca^2+^-dependent chloride secretion channel protein, but it does not contain traditional transmembrane domains and it is found secreted as a globular protein. The VWA domain of secreted CLCA1 modulates the activity of CaCC TMEM16A by directly engaging the channel at the cell surface to increase Ca^2+^-dependent chloride currents in human cells.^4^^,^^5^ However, it remains intriguing but unknown whether such process involves a different channel protein or accessary protein(s) such as the CFTR protein,^8^^,^^52^ the β-subunit KCNMB1,^24^ β_4_ integrin,^75^ or β2-integrins LFA-1 and Mac-1.^90^ Combination of CLCA1 with these different accessary proteins may explain the observations from different cancers and mouse respiratory disease models that we discussed in this review. Therefore, mechanistic studies using mCLCA1-deficient mice, cells lines, or specific CLCA1 blocking agents together with modern techniques may help us recognize the importance of this mysterious regulator of CaCCs in human pathobiology. Yet, as discussed above, there are eight mouse mCLCA genes and four human hCLCA genes. These multiple CLCA family members in humans or mice may exhibit certain levels of functional redundancy in the pathogenesis of human diseases or in mouse disease models, which can make it difficult to study the precise functions of CLCA1 in humans and mice even with the development of mouse models mCLCA1 deficiency. Nevertheless, further studies from cultured cells and disease models may help explore the mechanisms by which this secreted protein modulates chloride channels, goblet cell hyperplasia, and innate immune responses.

**Table 1:** Roles of CLCA1 in different diseases.

Disease type	Disease name	Proposed Function of CLCA1	Possible Mechanism of CLCA1
	Asthma	Involves in mucin hypersecretion and airway hyper-responsiveness^38^^,^^39^	Involves in Th2 responses^34^^,^^35^
	COPD	Involves in mucin hypersecretion^44^	Activates the MAPK13 pathway^44^
**Respiratory diseases**	Cystic fibrosis	Contributes to CaCC-mediated Cl^−^ and fluid secretion^46^	Mediates the CF-modulatory role of CaCCs^46^
	Pneumonia	Impacts immune response and mucus production^54^^,^^56^	Modulates the expression of CXCL-1 and IL-17^54^
	Mucin synthesis	Involves in mucin hypersecretion^57^	Activates the STAT6/SPDEF pathway^58^
	Ovarian cancer	Promotes cancer metastasis^68^	
**Cancers**	Colorectal cancer	Suppresses tumor growth^71^	Inhibits the Wnt/β-catenin pathway and EMT process^71^
	Lung cancer	Promotes cancer cell migration and metastasis^74^^,^^75^	Binds to the β_4_ integrin and activates the FAK/ERK pathway^75^
	Murine DSS-induced colitis	Involves in the modulation of cytokine responses^16^^,^^79^	Modulates the expression of CXCL-1 and IL-17^16^^,^^79^
**Gastrointestinal diseases**	CF intestinal disease	Relates to mucous properties and secretion^81^	Regulates goblet cell hypertrophy and hyperplasia^82^^,^^83^
	Ulcerative colitis	Relates to mucous properties and secretion^77^^,^^84^	
	Parasitic nematode infection	Impacts immune response and mucus production^86^	Involves in type 2 responses^86^
**Lymphatic system**		Coordinates lymph node remodeling^89^, ^90^	Serves as a lymphatic endothelial surface ligand^90^

CLCA1: calcium-activated chloride channel regulator 1; COPD: chronic obstructive pulmonary disease; DSS: dextran sodium sulfate; CF: cystic fibrosis; CaCC: calcium-activated chloride currents; EMT: epithelial–mesenchymal transition; FAK: focal adhesion; ERK: extracellular signal-regulated kinase; CXCL-1: C-X-C motif chemokine ligand 1; STAT6: signal transducer and activator of transcription 6; SPDEF: sterile alpha motif [SAM] domain-containing prostate-derived Ets transcription factor; EMT: epithelial-mesenchymal transition.

## Authors’ contributions

CLL drafted the manuscript; GPS critically revised the manuscript before submission. All authors read and approved the final manuscript.

## Ethics approval and consent to participate

Not applicable.

## Declaration of competing interest

The authors declare no conflicts of interest and agree to publish the work.
